# CreER activation transiently disrupts angiogenesis by reducing proliferation and promoting apoptosis in vascular endothelial cells

**DOI:** 10.1007/s10456-026-10040-0

**Published:** 2026-05-05

**Authors:** Elena Ioannou, Mengmeng Dong, Victoria S. Rashbrook, Christiana Ruhrberg, Martina Rudnicki

**Affiliations:** 1 UCL Institute of Ophthalmology, University College London, 11-43 Bath Street, London, EC1V 9EL UK; 2 Present Address: Department of Physiology, Anatomy and Genetics, University of Oxford, Sherrington Building, Sherrington Rd, Oxford, OX1 3PT UK

**Keywords:** Angiogenesis, Organ vascularization, Retina, Endothelial cell, CreER toxicity

## Abstract

Tamoxifen-inducible gene targeting in mice with estrogen receptor-dependent Cre (CreER) recombinase has enormously advanced vascular biology research. However, CreER activation under the control of vascular endothelial promoters causes off-target effects that impair the vascularization of the retina, a widely used model to study the molecular and cellular mechanisms of angiogenesis. Although ubiquitously expressed CreER is also used to study retinal angiogenesis, it remains unknown whether it causes similar or more severe off-target effects compared to endothelial-selective CreER activation. Moreover, the molecular and cellular processes disrupted by CreER activation in endothelial cells remain to be identified. Here, we demonstrate that ubiquitous CreER activation in postnatal mice decreases body growth and impairs retinal angiogenesis. We further show that CreER activation slows endothelial cell proliferation and promotes apoptosis concomitant with p21/CDKN1A upregulation in the angiogenic retina, although retinal vasculature progressively normalizes. By contrast, CreER activation in quiescent adult retinal vasculature did not induce endothelial cell apoptosis or p21 activation. Altogether, our findings indicate that ubiquitous promoters should be avoided to drive CreER expression when studying gene function during retinal angiogenesis, and that CreER activation controls, although essential to account for endothelial apoptosis and proliferation defects in short-term studies, may be less critical for adult vascular studies.

**Supplementary Information:**

The online version contains supplementary material available at 10.1007/s10456-026-10040-0.

## Introduction

Cre recombinase-mediated gene ablation offers an invaluable genetic tool to uncover molecules that regulate vascular biology in the mouse, the major mammalian model system [1]. Commonly, this method involves the use of Cre fused to a human estrogen receptor variant (ER); this fusion protein, termed CreER, is retained in the cytoplasm until it binds 4-hydroxy-tamoxifen (4-OHT), thereby enabling its nuclear translocation to induce gene recombination [2]. Administering 4-OHT or its precursor tamoxifen to promote Cre-mediated recombination therefore allows to control the time of deletion for genes that are flanked by loxP sites (floxed). By activating CreER after birth in vascular endothelial cells, this method circumvents the lethality caused by ablating essential vascular genes in utero [3]. Accordingly, this method allows to uncover molecular pathways that contribute to the growth, remodeling and function of postnatal blood vessels [1, 4]. For example, the CreER system has been used to demonstrate the role of vascular endothelial growth factor (VEGF) and notch signaling in postnatal angiogenesis [5–11].

Despite its usefulness for genetic investigation, a prior study demonstrated that CreER activation in endothelial cells with the commonly used *Cdh5*-CreER [11] or *Pdgfb*-CreER [12] transgenes can impair retinal angiogenesis [13]. However, it has not yet been addressed whether activation of ubiquitously expressed transgenes such as *Cagg*-CreER or *Rosa26*-CreER cause similar or worse detrimental effects during angiogenesis, even though these transgenes have also been used to delete genes in endothelial cells or cells in their environment (e.g., [9, 14–16]). Moreover, the mechanism by which CreER activation impairs angiogenesis remains unknown. Prior studies in non-endothelial cell types have suggested that Cre recombinase has toxic effects because it recognizes endogenous genomic DNA sequences that resemble the engineered loxP sequences used to mark genes for recombination [17–19]. Thus, off-target endonuclease activity directed at pseudo-loxP sites has been linked to DNA breaks, chromosomal aberrations, reduced cell proliferation, increased apoptosis and pro-inflammatory responses in non-endothelial cell types [4, 20, 21]. However, whether CreER activation also perturbs endothelial cell health in this manner has not been addressed. Moreover, it remains unknown whether the off-target effects of endothelial CreER activation are restricted to angiogenic endothelium or similarly affect the quiescent adult endothelium.

Considering that the mouse perinatal retina is widely used to identify molecules that regulate postnatal angiogenesis [22–24], we have used this model to investigate the emergence, mechanisms and resolution of off-target effects of CreER activation in endothelial cells. In the mouse, blood vessels emerge from vessels in the optic nerve head shortly after birth to form a superficial vascular plexus in the retina. Led by filopodia-studded tip cells, vessels grow forwards to cover the retina and fuse laterally, thereby establishing a planar network that is exquisitely suited for high-resolution imaging and quantification of angiogenic processes [3, 22, 24, 25]. Prior work showed that CreER activation in endothelial cells impaired both vessel extension and branching to significantly reduce vascular complexity as key angiogenic parameters in the retina [13].

Here, we show that CreER activation (but not tamoxifen or the vehicle used to administer tamoxifen) impairs retinal angiogenesis downstream of cell cycle stalling and increased apoptosis in retinal endothelial cells. Upregulation of the p21 cyclin dependent kinase inhibitor A1 (CDKN1A), which mediates DNA damage responses [26], was an early indicator of CreER-induced cellular stress in angiogenic endothelial cells. By contrast, CreER activation in quiescent endothelial cells did not upregulate p21, promote apoptosis or reduce vascular density. Our findings inform on confounding mechanisms that might skew data interpretation when using the CreER-loxP system to assess candidate angiogenesis regulators whilst also demonstrating that targeting of the adult vasculature does not induce similar off-target effects.

## Results

### Tamoxifen or its vehicle do not reduce weight gain or retinal angiogenesis in neonatal mice

**Fig. 1:**
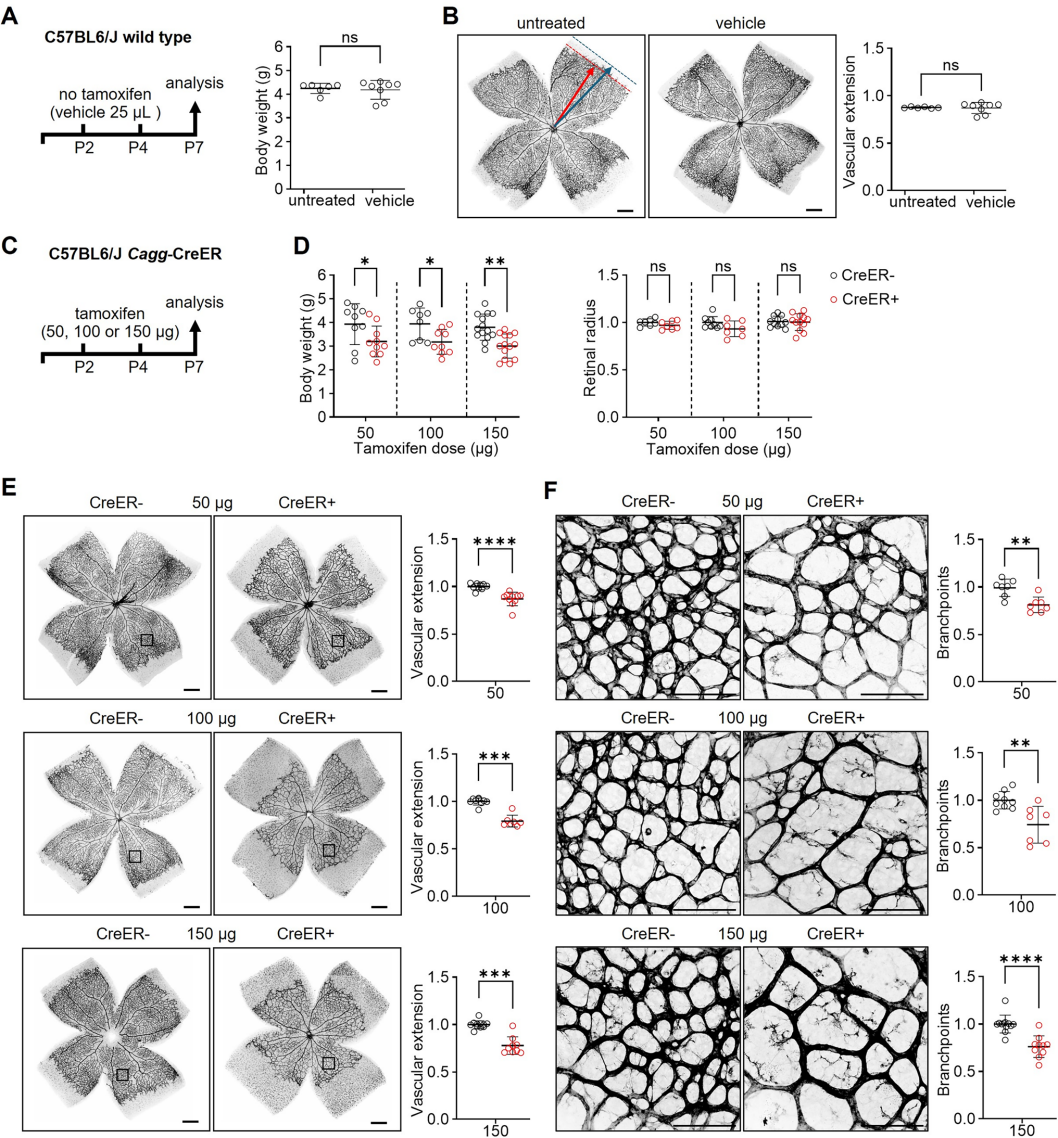
*Cagg*-CreER activation impairs weight gain and retinal angiogenesis in postnatal mice. **A** Schematic representation of the experimental setup and body weight measurement of P7 wild-type C57BL6/J pups treated with vehicle (25 μL vegetable oil, n = 8) on P2 and P4, compared to untreated controls (n = 6). **B** Representative images of P7 whole-mount retinas stained with IB4 from untreated and vehicle-treated pups, and quantification of vascular extension (n = 6-8 retinas per group). For each individual retina, vascular extension was measured as the ratio of the distance from the optic nerve head to the peripheral vascular front (red arrow and dotted line), relative to total retinal radius (blue arrow and dotted line). **C**-**F** Schematic representation of the tamoxifen treatment of *Cagg*-CreER+ and *Cagg*-CreER– pups (**C**), alongside (**D**) quantification of body weight and retinal radius on P7 following tamoxifen administration at the indicated doses on P2 and P4 (n = 7-14 pups per group). (**E**, **F**) Representative images of P7 CreER+ and CreER– whole-mount retinas stained with IB4 (shown in inverted grey scale), including quantification of vascular extension (**E**, n = 7-11 pups per group) and branchpoints (**F**, n = 7-12 pups per group). Values from CreER+ samples were normalized to the average of the values for the CreER– group. Black square boxes in (**E**) indicate regions between an artery and a vein shown at higher magnification in (**F**) and used for branchpoint analysis. Scale bars: 500 µm (vascular extension); 100 µm (branchpoints). Data are shown as mean ± SD. Each data point represents one mouse (body weight) or the average of four measurements from one retina from one mouse (retinal radius and vascular parameters). *P < 0.05; **P < 0.01; ***P < 0.001; ****P < 0.0001; ns: not significant (*P* > 0.05). Mann-Whitney U test

Vegetable oils are commonly used as vehicle for tamoxifen administration but can cause inflammation in adult mice when delivered via intraperitoneal injection [27]. To determine whether the intraperitoneal injection of vegetable oil as a vehicle affects growth or angiogenesis in postnatal mice, we delivered two 25 μL doses, one on perinatal day (P) 2 and one on P4 and examined both male and female C57BL6/J wild type pups on P7 in the absence of floxed target genes (Fig. 1A). We found that vegetable oil on its own did not affect pup weight gain or retinal vascular network extension when compared to untreated pups (Fig. 1A, B). As tamoxifen has known vascular effects [28] and can reduce body weight independently of CreER activation in adult mice [29], we also investigated the effect of tamoxifen in neonatal pups that lacked floxed target genes. For this experiment, we tested three tamoxifen doses commonly used for gene targeting in the neonatal period, 50, 100 or 150 µg (e.g., [14–16, 30]) by delivering them as 25 µL intraperitoneal injections in vegetable oil on P2 and P4. We found that neither the 50, 100 nor 150 µg tamoxifen doses affected body weight or vascular extension in male or female C57BL6/J pups at P7 (Fig. S1A).

### Ubiquitous CreER activation reduces weight gain and retinal angiogenesis in neonatal mice

Next, we compared tamoxifen-injected neonatal C57BL6/J mice carrying or lacking the *Cagg*-CreER transgene [31] without floxed genes to distinguish toxic effects of CreER activation from the effects of gene targeting. This transgene was selected because it is useful to determine how deleting genes in the environment of endothelial cells affects retinal vascular endothelial cells, for example, when studying how secreted factors modulate endothelial cell behavior [14, 16, 32, 33]. Thus, we delivered two doses of 50, 100 or 150 µg tamoxifen, one on P2 and one on P4, to C57BL6/J mice expressing or lacking *Cagg-*CreER and also lacking floxed target genes (Fig. 1C). Of note, the *Cagg-*CreER transgene was effectively induced already 24 h after the second tamoxifen injection, as demonstrated via activation of the floxed *Rosa26*^*tdTom*^ recombination reporter (Fig. S1B). At P7, we observed that male and female pups with activated CreER (CreER+) had lower body weight compared to control pups lacking CreER (CreER−) across all tamoxifen doses examined (Fig. 1D). By contrast, the retina radius was similar in pups with or without activated CreER at all three doses examined (Fig. 1D), which allowed us to compare the effect of tamoxifen-induced CreER activation on retinal angiogenesis in the absence of confounding effects of eye size. Both vascular network extension and retinal vascular branch density, key angiogenic parameters, were significantly reduced in retinas from CreER+ compared to CreER− mice at all three tamoxifen doses examined (Fig. 1E, F). Further, we found no evidence for treatment-by-sex interaction (χ^2^ = 2.61, df = 2, *P* = 0.27), suggesting similar effects of CreER activation on retinal vascularization in both sexes. The reduction in vascular extension was consistent but significantly less for 50 μg tamoxifen compared to 100 μg (*P* = 0.02) or 150 μg (*P* < 0.001). Notably, however, 150 μg tamoxifen did not further reduce vascular extension than 100 μg tamoxifen (*P* = 0.63), suggesting that toxicity reaches a plateau with 2 doses of 100 μg tamoxifen for *Cagg-*CreER activation. Moreover, the reduction in vascular branchpoints was similar across all three tamoxifen doses (dose comparison: 50 vs. 100, *P* = 0.48; 50 vs. 150, *P* = 0.63; 100 vs. 150, *P* = 0.94). Notably, the presence of loxP sites (introduced via the floxed *Rosa26*^*tdTom*^ reporter) did not ameliorate the detrimental effect of CreER activation on vascular extension (Fig. S1C).

Together, these findings demonstrate that a ubiquitously expressed CreER transgene used to target retinal angiogenesis, impairs retinal angiogenesis at the lowest dose examined and even when the genome contained engineered loxP sites as intended CreER targets. Additionally, ubiquitous *Cagg-*CreER activation impaired body growth, which was not seen with endothelial-selective CreER activation.

### CreER activation-induced retinal vascular defects recover over time

**Fig. 2:**
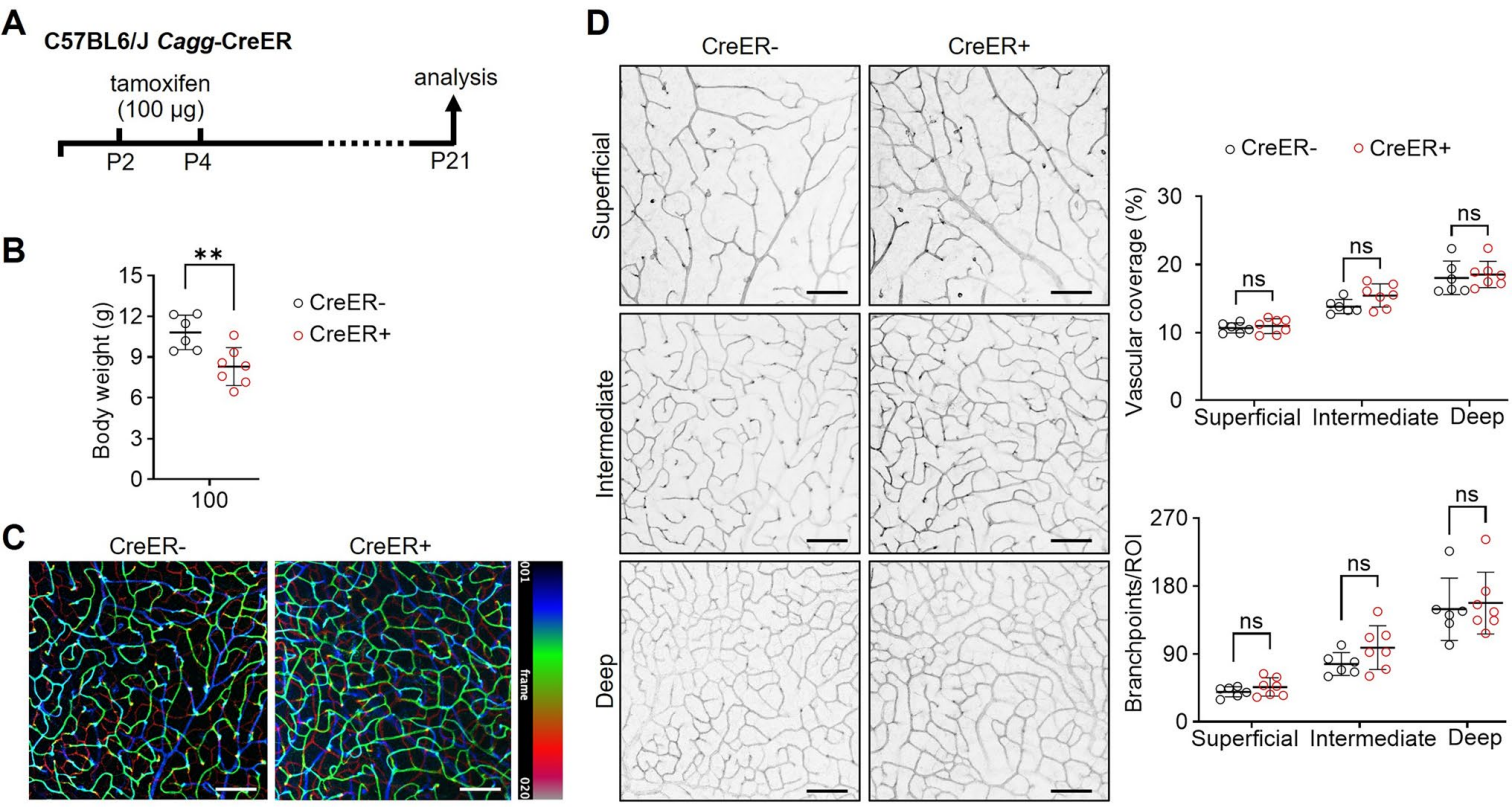
Recovery of retinal angiogenesis but not body weight defects after past CreER activation. **A**–**D** *Cagg*-CreER+ and *Cagg*-CreER− littermate pups on a C57BL6/J background were treated with 100 µg tamoxifen on P2 and P4 and examined on P21. **A** Schematic representation of the experimental setup. **B** Body weight (n = 6–7 per group). **C**, **D** Whole-mount retinas of CreER+ and CreER− pups were stained with IB4. **C** Representative projection of confocal z-stack images, with the vascular plexi resolved by pseudo-coloring according to retinal depth (20 z-slices; blue: superficial, green: intermediate, red: deep). **D** Representative images of the three IB4 + retinal plexi, (shown in inverted greyscale), extracted from the confocal z-projection shown in (**C**), and quantification of vascular coverage and branchpoints per region of interest (ROI) between an artery and vein for each plexus (n = 6–7 per group). Scale bars: 100 µm. Data are shown as mean ± SD. Each data point represents one mouse (body weight) or the average of four regions of interest (ROI) from one retina from one mouse (vascular measurements). **P < 0.01; ns: not significant (*P* > 0.05). Mann–Whitney U test.

In adult mice, tamoxifen is metabolized to 4-OHT, whereby both compounds have a serum half-life of less than 10 h and are barely detectable by 24–48 h after administration [34, 35]. We therefore examined whether tamoxifen discontinuation allowed body weight and retinal angiogenesis to recover from the defects caused by earlier tamoxifen-induced CreER activation after pups had received two doses of 100 μg tamoxifen, one on P2 and another one on P4. This dose was chosen because it disrupts both vascular extension and branching not only with the ubiquitous transgene employed in our study but also with two endothelial-specific transgenes used previously [13]. We examined retinal vasculature at P21, more than two weeks after tamoxifen had been discontinued (Fig. 2A). At this time, CreER+ mice still had significantly lower body weight compared to control littermates (Fig. 2B). We next acquired confocal z-projections that spanned the depth of all three vascular plexi of the maturing retinal vasculature (Fig. 2C) and grouped individual slices of the z-projection according to their representation of each plexus (Fig. 2D). Although the complexity of the superficial vascular plexus was impaired at P7 (see Fig. 1E and F), we observed normal vascular coverage and branchpoint complexity in the superficial retinal vascular plexus at P21 (Fig. 2D). Moreover, the deep plexus, which sprouts from the superficial plexus after P7, and the intermediate plexus, which forms from the deep plexus after P12 [23], also had normal vascular complexity (Fig. 2D). In order to refine our understanding of the time frame in which vascular normalization occurred, we first examined retinal vasculature at P15 (Fig. S2A), i.e., 11 days after the last tamoxifen injection. We found that vascular coverage was still reduced at this time and also observed vascular abnormalities in 4/6 retinas analyzed (Fig. S2A–C). We then assessed retinal vasculature at P17, i.e., 13 days after the last tamoxifen injection. Vascular abnormalities were observed in 2/3 retinas from CreER+ pups analyzed at this timepoint, but vascular coverage was now similar to retinas of CreER− littermates (Fig. S2A–C). Together, these findings suggest that CreER activation impairs retina vascularization for a prolonged period, but that the vasculature can progressively normalize over time after tamoxifen discontinuation. By contrast, the body weight reduction persisted beyond the period in which vascular normalization occurred (Fig. 2B).

### CreER activation impairs cell cycle progression during retinal angiogenesis

**Fig. 3:**
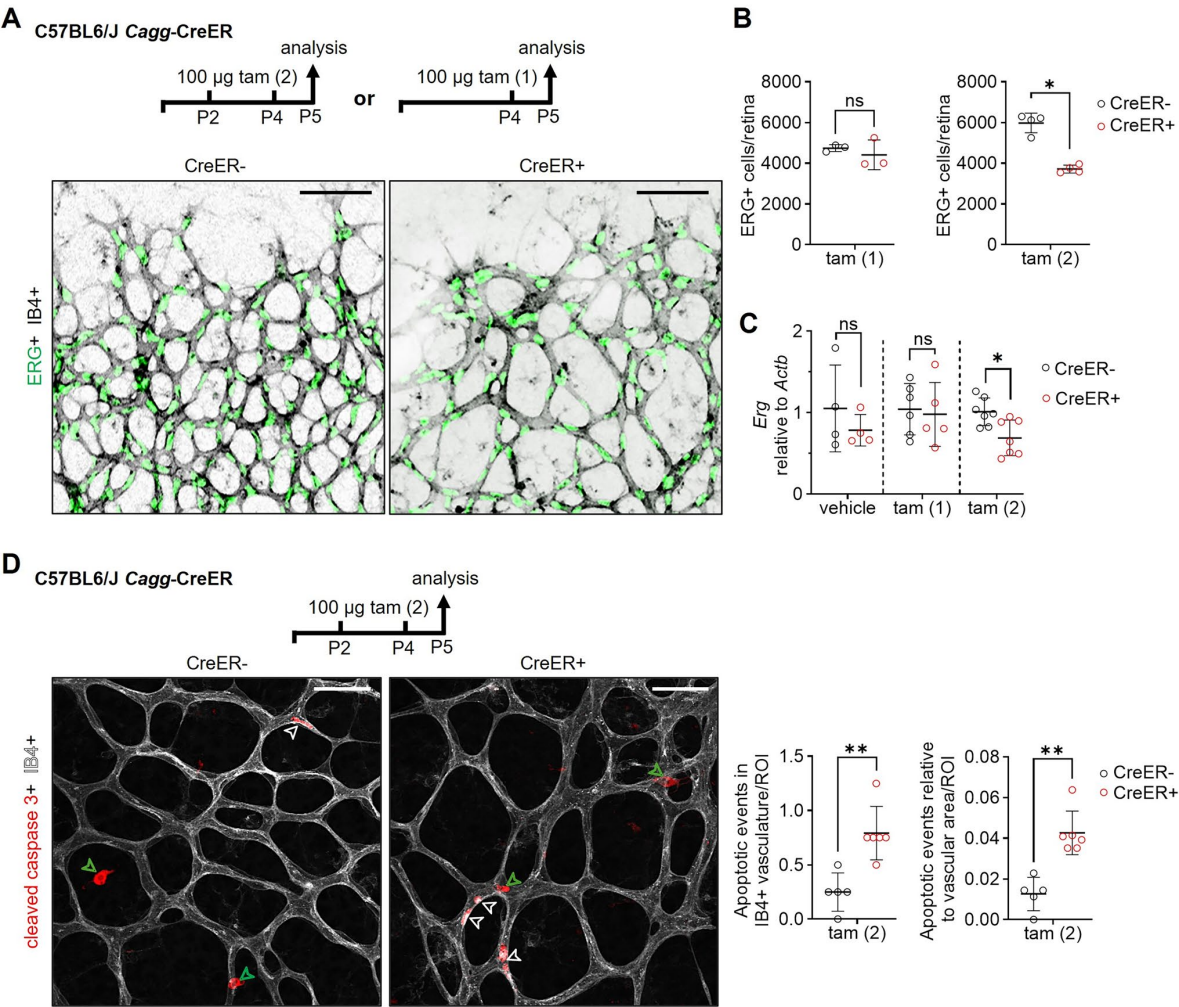
*Cagg*-CreER activation reduces endothelial cell proliferation and upregulates p21. **A**–**D** *Cagg*-CreER+ and *Cagg*-CreER− littermate pups on a C57BL6/J background were treated with 100 µg tamoxifen on P2 and P4, referred to as the tam (2) condition, or 2 doses of vehicle at similar timepoints, or with 100 µg tamoxifen on P4 only, referred to as the tam (1) condition, and then analyzed on P5; schematic representations of the experimental setup are shown in each panel. One retina from each mouse was used for staining whereas the other retina was used for gene expression analysis. Scale bars: 100 µm. **A** P5 retinas were labeled with IB4 (shown in inverted greyscale) and stained for ERG (shown in green) and (**B**) the number of ERG + endothelial cells per retina. **C** Quantification of *Erg* mRNA levels relative to *Actb*. **D** P5 retinas were labeled with IB4 (shown in greyscale) and stained for cleaved caspase 3 (shown in red), including quantification of cleaved caspase 3 + cells (apoptotic events) detected in IB4+ vasculature and the number of apoptotic events detected in IB4 + vasculature corrected by the vascular density in each region of interest (ROI). Open white arrowheads, cleaved caspase 3 positive endothelial cells. Open green arrowheads, cleaved caspase 3 outside vessels. Data are shown as mean ± SD. Each data point represents one retina from one mouse (n = 4–8 per group). Gene expression results are reported as fold change relative to the average of the CreER− group. *P < 0.05; **P < 0.01; ns: not significant (*P* > 0.05). Mann–Whitney U test

To understand the mechanisms underlying CreER-induced angiogenesis defects, we administered one or two doses of 100 μg tamoxifen, referred to as 100 μg tam (1) and 2 × 100 μg tam (2), respectively, and then examined retinas 24 h after a single or the second tamoxifen injection for altered endothelial cell behavior that might explain subsequent angiogenesis defects (Fig. 3A). The body weight of CreER− and CreER+ pups was still similar at this early time point after CreER activation (Fig. S3A, B). The quantitative analysis of retinas stained for IB4 together with an antibody for the endothelial-specific transcription factor ERG [36] revealed 35% fewer ERG+ endothelial cells in CreER+ compared to CreER− retinas after the second tamoxifen dose, but not after a single tamoxifen dose (Fig. 3A, B). Consistent with these observations, quantitative RT-PCR analysis of retinas from the opposing eye of the same mice showed a 30% decrease of *Erg* transcript levels after two but not one tamoxifen dose or after vehicle treatment (Fig. 3C).

To examine whether the reduction in ERG+ cells resulted from increased cell death, we assessed P5 retinas after tamoxifen treatment on P2 and P4 by staining for IB4 together with the antibody for the apoptosis marker cleaved caspase 3 (e.g., [37]). Using this method, we observed a three-fold average increase in endothelial apoptosis in CreER+ compared to CreER− retinas, although apoptotic events were rare, even when corrected by the vascular density in each region of interest for both conditions (Fig. 3D). By contrast, the number of non-endothelial cells that were positive for activated caspase 3 was similar in CreER+ compared to CreER− retinas, although the ubiquitous *Cagg*-CreER transgene should have been activated in all retinal cell types (Fig. S3C). These findings suggest that CreER activation enhances apoptosis of endothelial cells.

**Fig. 4:**
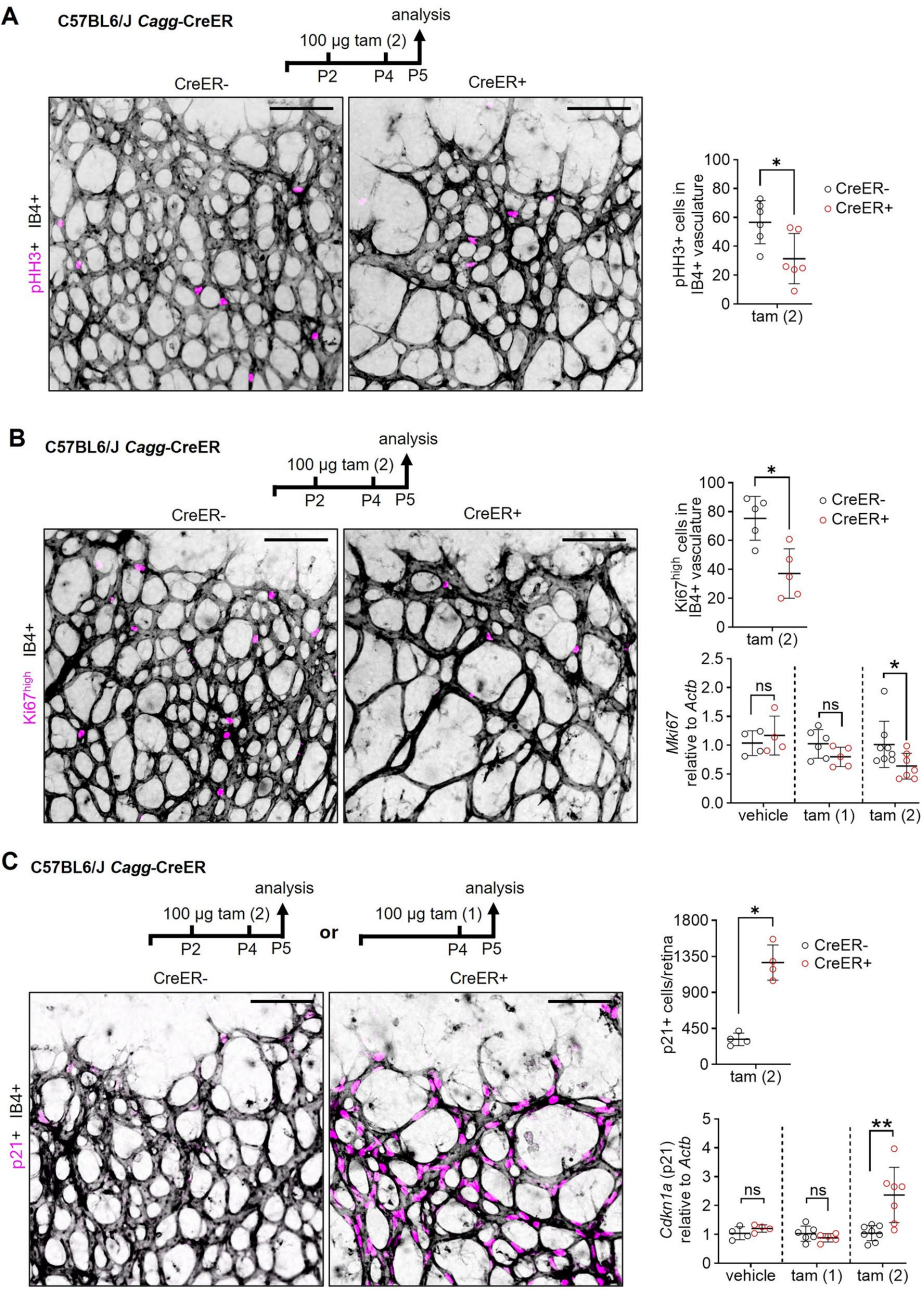
*Cagg*-CreER activation reduces endothelial cell proliferation and upregulates p21. **A**–**C** *Cagg*-CreER+ and *Cagg*-CreER− littermate pups on a C57BL6/J background were treated with either 100 µg tamoxifen on P2 and P4, referred to as the tam (2) condition, or 2 doses of vehicle at similar timepoints, or with 100 µg tamoxifen on P4 only, referred to as the tam (1) condition, stained for IB4 (shown in inverted grey scale) together with antibodies for the indicated markers (shown in magenta) and then analyzed on P5; schematic representations of the experimental setup are shown in each panel. One retina from each mouse was used for staining whereas the other retina was used for gene expression analysis. Scale bars: 100 µm. **A** P5 retinas labeled with IB4 and stained for pHH3 including quantification of pHH3 + cells detected in IB4+ vasculature. **B** P5 retinas labeled with IB4 and stained for Ki67 including quantification of Ki67^high^ cells detected in IB4+ vasculature 24 h after the second tamoxifen injection, and quantification of mRNA levels for *Mki67* (encoding Ki67) relative to *Actb*. **C** P5 retinas labeled with IB4 and stained for p21, including quantification of p21+ cells per retina and quantification of mRNA levels of *Cdkna1* (encoding p21) relative to *Actb*. Data are shown as mean ± SD. Each data point represents one retina from one mouse (n = 4–8 per group). Gene expression results are reported as fold change relative to the average of the CreER− group. *P < 0.05; **P < 0.01; ns: not significant (*P* > 0.05). Mann–Whitney U test.

To determine whether decreased endothelial cell proliferation additionally contributed to the reduced number of ERG+ cells after CreER activation, we examined P5 retinas after tamoxifen treatment on P2 and P4 by staining for IB4 together with two proliferation markers, the mitotic marker phospho-histone H3 (pHH3), which is detected in cells during the late G2 and M phases (but not in telophase; [38]), and the proliferation marker Ki67, which is expressed during all active cell cycle phases [39]. Quantitative analysis of retinas stained with an antibody for pHH3 revealed 45% fewer pHH3+ endothelial cells in CreER+ compared to CreER− retinas (Fig. 4A). For Ki67 staining, we compared two commercially available antibodies (Fig. S3D, E). In agreement with published results [40], one antibody detected a large number of endothelial cells in the P5 retina, including Ki67^high^ and Ki67^low^ cells, whereas the other one [41] detected only Ki67^high^ cells in the retina (Fig. S3D). The latter antibody was suitable for double labelling with the antibody we used for pHH3, and double labelling showed that most Ki67^high^ cells were also pHH3+ (Fig. S3D, E); accordingly, this antibody combination was chosen for subsequent analysis. Agreeing with the reduced number of pHH3+ endothelial cells, we detected a 49% reduction in Ki67^high^ endothelial cells in CreER+ compared to CreER− retinas (Fig. 4B), indicating that fewer endothelial cells were actively cycling after CreER activation. Contrasting the findings for endothelial cells, the number of Ki67^high^ non-endothelial cells was similar and the number of pHH3+ non-endothelial cells was increased in CreER+ versus CreER− retinas (Fig. S3F, S4). Together, these findings suggest that a subset of non-endothelial cells had arrested in mitosis after CreER activation, which was not seen for endothelial cells, which instead had stopped cycling without a selective arrest in mitosis.

As the above data suggest that CreER activation stalls endothelial cell cycle progression, we examined retinas for the expression of p21, which is a known regulator of both DNA replication and DNA damage repair [26] and was previously shown to be upregulated after *Cdh5*-CreER activation [40]. Thus, we labelled whole-mount retinas for IB4 together with an antibody specific for p21 24 h after the second of two tamoxifen injections (Fig. 4C). We found that the number of p21+ endothelial cells increased fourfold in CreER+ compared to CreER− retinas (Fig. 4C). Despite ubiquitous *Cagg*-CreER activity in endothelial and non-endothelial cells in the retina, p21 upregulation was confined to the IB4+ area (Fig. S4). Quantitative RT-PCR analysis corroborated that p21 was significantly upregulated in CreER+ compared to CreER− retinas after two doses of tamoxifen but not in P5 retinas of vehicle-treated mice (Fig. 4C).

We also observed an increased number of p21+ endothelial cells in CreER+ compared to CreER− P5 retinas after a single tamoxifen dose on P4 (but not with vehicle; Fig. S5A, B). However, the increase in p21+ endothelial cells was lower than after two doses of tamoxifen and was not reflected in transcriptional upregulation (Fig. 4C). This finding suggests that p21 upregulation initially occurred independently of transcriptional induction, consistent with prior knowledge that post-transcriptional mechanism regulate p21 protein stability by countering ubiquitin-mediated degradation pathways [26]. This early response identifies p21 as a sensitive marker of CreER-mediated cellular stress in endothelial cells. Moreover, the increased number of p21+ endothelial cells in CreER+ compared to CreER− retinas persisted to P15 and P17 (Fig. S5C, D), when retinal vasculature was still undergoing normalization.

Together, these results imply that p21 protein levels increase rapidly in endothelial cells following CreER activation and precede overt vascular defects, such as fewer ERG+ cells, reduced vascular extension and branching, and this p21 upregulation is associated with a period of increased endothelial cell apoptosis and reduced proliferation followed by vascular normalization.

### CreER-induced p21 upregulation occurs in angiogenic, not quiescent, endothelium

**Fig. 5:**
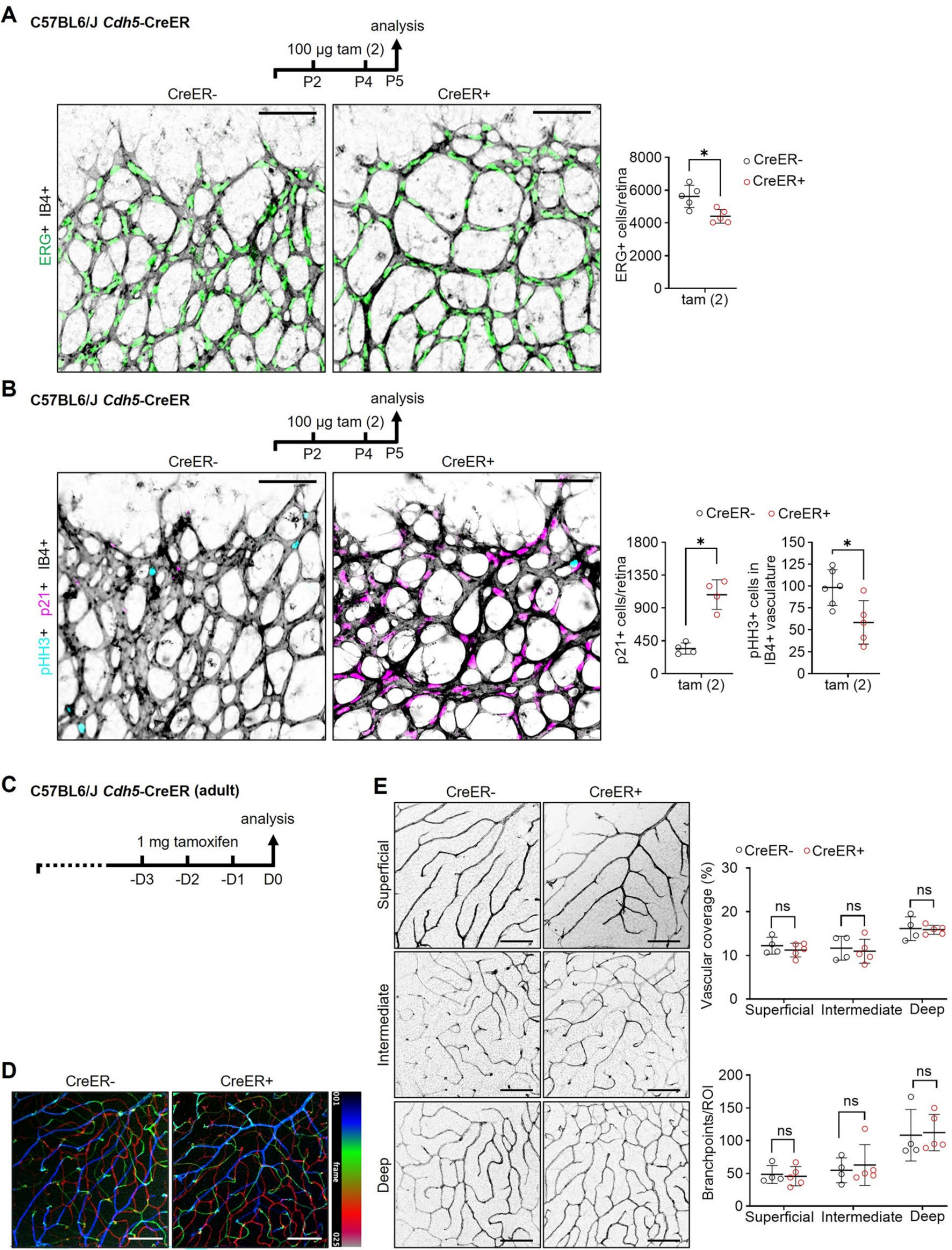
Reduced vascular growth and p21 upregulation in angiogenic but not in quiescent endothelium after *Cdh5*-CreER activation. **A**, **B** *Cdh5*-CreER+ and *Cdh5*-CreER− littermate pups on a C57BL6/J background were treated with 100 µg tamoxifen on P2 and P4, referred to as the tam (2) condition, and their retinas were analyzed on P5. Schematic representations of the experimental setup are shown in each panel. P5 retinas labeled with IB4 (shown in inverted greyscale) and stained for ERG (shown in green), including quantification of ERG+ endothelial cells per retina (**A**) or stained for p21 (shown in magenta) and pHH3 (shown in cyan), including quantification of p21+ cells per retina and pHH3+ cells detected in IB4+ vasculature per retina (**B**). Scale bars: 100 µm. **C**–**E** Schematic representation of the experimental setup for analysis of adult *Cdh5*-CreER+ and *Cdh5*-CreER− mice on a C57BL6/J background; mice were treated with 1 mg tamoxifen daily for three consecutive days (D; -D3, -D2 and -D1) and analyzed 24 h after the last injection (D0). **D** Representative projection of confocal z-stack images from whole-mount retinas of CreER+ and CreER− mice stained with IB4; with vascular plexi resolved by pseudo-coloring according to retinal depth (25 z-slices; blue: superficial, green: intermediate, red: deep). **E** Representative images of the three IB4+ retinal plexi, shown in inverted greyscale, extracted from the confocal z-projection shown in (**D**) including quantification of vascular coverage and branchpoints per region of interest (ROI) between an artery and vein in each plexus (n = 4–5 per group). Scale bars: 100 µm. Data are shown as mean ± SD. Each data point represents one ROI for one retina from one mouse. *P < 0.05; ns: not significant (*P* > 0.05). Mann–Whitney U test

To determine whether CreER-induced cell cycle effects were also observed with endothelial-selective CreER activation, we next examined mice carrying the *Cdh5*-CreER transgene, for which endothelial CreER off-target effect was first described [13]. Similar to *Cagg*-CreER mice, P5 CreER+ mice, which had received two tamoxifen injections on P2 and P4, had 22% fewer ERG+ cells, a 59% decrease in the number of pHH3+ endothelial cells, and a threefold increase in the number of p21+ endothelial cells compared to CreER-negative littermates (Fig. 5A, B). In contrast, the retinas of 5/5 adult CreER+ mice treated with three consecutive tamoxifen injections (1 mg per injection every 24 h) exhibited no obvious changes in vascular density compared to CreER− littermates, and neither p21+ endothelial cells nor cleaved caspase 3+ endothelial cells were observed (Fig. 5C–E). Similar findings were made when CreER was activated in *Cagg*-CreER-expressing adult mice (data not shown). Together, these results suggest that both ubiquitous and endothelial-specific CreER activation at the postnatal stage impair endothelial cell proliferation following p21-induced cell cycle arrest, likely as a response to off-target Cre endonuclease activity.

## Discussion

CreER-loxP mutagenesis in the mouse has considerably advanced our understanding of vascular biology and remains the primary approach for investigating angiogenesis regulators. For example, the spatiotemporal control of gene recombination in the developing retina has enabled pivotal insights into key regulatory pathways such as VEGF, TGFβ and notch signaling, as well as their intricate cross talk during angiogenesis (e.g., [5, 7, 8, 11, 42]). However, recent studies have highlighted a significant caveat with this widely used system. Specifically, we and others have shown that endothelial cells in the angiogenic retina are highly susceptible to the toxic effects of CreER activation independently of targeted gene deletion [13, 40], thereby creating the risk that the interpretation of angiogenesis effects in knockdown studies may be inaccurate unless CreER off-target effects are accounted for. Here, we demonstrate that CreER activation does not affect quiescent endothelium but that Cre-induced vascular defects in the angiogenic retina are attributable to both increased apoptosis and reduced endothelial proliferation. Importantly, our findings also reveal that CreER-induced cellular stress in proliferating endothelial cells is not mitigated by the presence of loxP sites but that the retinal vasculature normalizes over time after tamoxifen withdrawal.

Ubiquitously expressed transgenes have been used to investigate the angiogenic effects of molecules expressed in endothelial cells (e.g., [9, 14–16, 33]). These transgenes are also valuable for reporting on angiogenic requirements of molecules secreted by other cell types in the vascular environment that affect endothelial cell behavior. While expected, our study shows that CreER activation has toxic effects on endothelial cells also when using a ubiquitous promoter, thereby reinforcing the importance of considering CreER-associated toxicity as a confounding variable in angiogenesis studies. Furthermore, we have made two additional notable observations, which suggest that endothelial-selective CreER activation should be prioritized when investigating potential angiogenesis regulators in endothelial cells, in order to minimize systemic confounding factors that arise from using a ubiquitously expressed CreER transgene. First, we detected reduced postnatal weight gain following *Cagg*-CreER activation across all tamoxifen doses tested, which was not observed for endothelial-specific CreER activation [13], and this effect persisted more than two weeks after tamoxifen discontinuation, when retinal vascular density had normalized. Widespread CreER activation therefore imposes a broader burden on early postnatal development that cannot be attributed to the effects on endothelial cells alone and suggests that ubiquitously expressed CreER should be avoided for CreER expression when cell-type specific CreER methods are available. Second, activating a ubiquitously and highly expressed CreER transgene impaired vascular extension even at the lowest tamoxifen dose tested (50 µg), which falls into the lower range commonly reported in retinal angiogenesis studies. Nevertheless, the use of ubiquitous promoters to drive CreER expression remains necessary for targeting candidate pro-angiogenic modulators expressed by non-endothelial cells and will therefore require appropriate controls to account for off-target effects in endothelial cells [4].

We also found that the repeated delivery of even low doses of tamoxifen to activate CreER becomes increasingly toxic and therefore does not provide a reliable strategy to mitigate CreER-associated toxicity. Under our experimental conditions, a single tamoxifen injection had no detectable impact on retinal endothelial cell number 24 h later; however, two injections caused a ~ 35% reduction in endothelial cell density 24 h after the second injection. Notably, the most commonly employed protocol for gene targeting in retinal angiogenesis studies involves three or four consecutive tamoxifen injections [3]. Our findings suggest that such repeated injections may exacerbate CreER-activation associated off-target effects beyond those we have observed with two injections, although the magnitude of the effect will likely also depend on CreER expression levels. In this context, a better understanding of the molecular mechanisms that underlie CreER toxicity for endothelial cells may help to identify dosing thresholds that have fewer off-target effects without compromising recombination of floxed target genes.

To determine which angiogenesis-relevant endothelial cell functions are affected by CreER activation, we considered previous reports showing that CreER activation lowers proliferation rates in mouse embryo fibroblasts and mouse keratinocytes in vitro [21] and promotes apoptosis of keratinocytes in vivo [43]. Of note, we established that vascular defects can be detected earlier than previously reported [13]. Specifically, we observed a lower number of ERG+ cells already on P5, two days before obvious angiogenesis defects were detected on P7. By staining for cleaved caspase 3, Ki67 and pHH3, we attributed this early growth defect after CreER activation to both increased apoptosis and reduced endothelial cell proliferation. By contrast, CreER activation in the quiescent adult vasculature did not affect vascular density, thereby suggesting that the angiogenic vasculature, which is highly proliferative, is more susceptible to CreER toxicity. Consistent with our findings in retinal endothelium, a prior study using a *Cx3Cr1*-CreER transgene demonstrated that CreER activation adversely affected microglia proliferation and increased microglial cell death in early postnatal brains, whereas no change in the microglia number was detected when *Cx3Cr1*-CreER was activated in adult mice [44]. Similarly, activating the ubiquitous *Rosa26*-CreER transgene reduced the proliferation and increased the apoptosis of immature hematopoietic cells in the thymus and spleen of adult mice [45], further reinforcing the notion that rapidly proliferating cells are more vulnerable to the toxic effects of CreER activation.

The cyclin-dependent kinase inhibitor p21 is a well-known regulator of cell cycle progression and effector that links the cell cycle to the DNA damage response [26]. According to our results, and those reported in a prior study [40], p21 upregulation is an early response to CreER activation during retinal vascularization. Although our experiments do not directly demonstrate causality between DNA damage, reduced endothelial proliferation and increased apoptosis, the observed p21 upregulation is consistent with cell cycle arrest in response to DNA damage. Considering that pseudo-loxP sites are unpaired, their cleavage is expected to cause single stranded breaks [17]. In non-proliferative cells, such breaks can be readily repaired using the opposing DNA strand as a template. By contrast, the single-stranded DNA present during S-phase is likely more vulnerable to damage and would require non-homologous end joining for repair. Supporting a model in which cells in S-phase are particularly vulnerable to CreER toxicity due to DNA damage, CreER activation did not upregulate p21 in endothelial cells of the quiescent retinal vasculature. It is therefore conceivable that CreER-induced p21 upregulation, secondary to DNA damage, may transiently stall cell cycle progression to allow for single stranded DNA repair. We therefore hypothesize that p21 contributes to vascular normalization by imposing a temporary pause in DNA synthesis or facilitating the elimination of damaged endothelial cells by apoptosis to pave the way for the progressive vascular normalization we observed more than two weeks after tamoxifen discontinuation. To validate this hypothesis, future work on retinal angiogenesis that combines CreER induction with a p21 knockout would be required to determine whether p21 is functionally required for CreER-induced effects on endothelial cell proliferation and apoptosis and whether it is essential for subsequent vascular recovery.

Notably, p21 upregulation was restricted to endothelial cells in the retina, not only after endothelial-selective CreER activation but also after ubiquitous CreER activation . Consistent with a protective role for p21 in promoting vascular resilience to CreER-induced stress, analysis of pHH3 and Ki67 staining in the retina revealed fewer cycling endothelial cells after CreER activation, without evidence of mitotic arrest. In contrast, a large number of non-endothelial cells, which showed no p21 upregulation, appeared to have stalled in mitosis, potentially indicating a cell cycle block. Importantly, p21 upregulation in retinal endothelial cells was detectable as early as 24 h after a single tamoxifen dose and preceded the detection of effects on cell cycle progression. These findings raise the question why endothelial cells in the retina may be uniquely primed to mount a rapid p21-mediated response to CreER activation. A plausible explanation might arise from the prior finding that p21 serves a physiological function in retinal vascular development. Specifically, it has been reported that p21 is selectively upregulated in a subset of endothelial cells at the vascular front to balance endothelial cell sprouting and proliferation in the context of high mitogenic stimulation [46]. It is therefore conceivable that CreER-mediated p21 upregulation and its associated function in regulating endothelial cell proliferation co-opts a physiological mechanism that normally operates at the angiogenic front at low levels, and thus serendipitously confers vascular resilience to the effects of CreER toxicity.

In summary, our results support the concept that CreER toxicity is a significant confounder in studies of postnatal angiogenesis and reveal that these effects also occur when a ubiquitously expressed transgene is employed to target endothelial cells. These findings emphasize the need for tamoxifen-activated CreER controls to disentangle CreER-induced effects from gene-specific phenotypes due to loxP-mediated recombination, and, in the future, should be extended from the retina to other organ contexts. Moreover, it may be interesting to investigate the extent of CreER toxicity in genetic backgrounds different to C57BL6/J, which we have chosen for our study due to its predominant use in gene targeting studies. Although our evidence suggests that quiescent vasculature in adult animals appears less affected by CreER toxicity, further work is also required to understand implications for mouse models of adult neovascular diseases, in which endothelial cell proliferation is reactivated, for example, in choroidal neovascular disease, tumor angiogenesis, or ischemic vascular diseases such as peripheral artery disease or myocardial infarction.

## Methods

### Animals

All procedures were conducted in accordance with the Institutional Animal Welfare Ethical Review Body and the United Kingdom Home Office guidelines under the Animals (Scientific Procedures) Act 1986. All mice were bred at the UCL Institute of Ophthalmology’s Biological Resources Unit and housed in individually ventilated cages at 20–24 °C ambient temperature, 55 ± 10% relative humidity, and a 12-h light/dark cycle. All mice had access to a standard diet and water ad libitum. In this study, Tg(CAG-cre/Esr1*)5Amc (*Cagg*-CreER) (MGI:212,767) [31] or Tg(Cdh5-cre/ERT2)#Ykub (*Cdh5*-CreER) (MGI:5,705,396) [11] males on a C57BL6/J background were crossed with wild-type females on a C57BL6/J background to generate mice carrying or lacking ubiquitous and endothelial cell-specific tamoxifen-inducible CreER, respectively. In some experiments, *Cagg*-CreER males were crossed with GT(*Rosa*) *26Sor*^*tm14(CAG−tdTomato)Hze*^ (*Rosa26*^*tdTom*^) (MGI:3,813,512) [47] females on a mixed genetic background (C57BL6/J:129/Sv).

### Whole-mount retina staining

Eyes were enucleated at the indicated time points and fixed in 4% formaldehyde in PBS for 10 minutes at room temperature (RT). Retinas were immediately micro-dissected and prepared for flat mounting by making four radial incisions. Dissected retinas were stored in 96-well plates at -20°C in 100% methanol until staining and were rehydrated in PBS before permeabilization. For cleaved caspase 3 staining, dissected retinas were fixed for a further 45 minutes in 4% formaldehyde in PBS at RT after flat mounting, then washed three times in PBS, and stored at 4°C in PBS containing 0.05% sodium azide until staining. For immunostaining, retinas were first permeabilized in PBS containing 0.5% Triton X-100 (PBST) for 30 minutes at RT. Retinas were then incubated for 1 hour at RT using either PBST containing 10% serum-free protein block (DAKO; X0909, Agilent) for tdTomato reporter staining together with biotinylated IB4, or for all other antibodies in a blocking solution comprised of PBST containing 10% heat-inactivated normal goat serum and 1% bovine serum albumin (Sigma-Aldrich). Retinas were incubated for 90 minutes at RT with the following primary antibodies diluted at 1:400 in blocking solution: anti-phospho-histone H3 (anti-phH3; Sigma-Aldrich, H9908), anti-Ki-67 (Abcam, ab16667), anti-ERG (Abcam, ab92513), anti-p21 (Abcam, ab188224). Alternatively, retinas were incubated overnight at 4°C with cleaved caspase 3 antibody (1:200; Cell Signaling Technology, 9661), anti-RFP for tdTomato detection (1:500, MBL Caltag-Medsystems, PM005) and biotinylated IB4 (1:200, Sigma-Aldrich, L2140). Following four 15-minute PBS washes, retinas stained with RFP antibody and/or biotinylated IB4 were incubated with Alexa Fluor® 647 AffiniPure® Fab Fragment Donkey Anti-Rabbit IgG (H+L) (1:200, Stratech Jackson, 711-607-003) for tdTomato staining and streptavidin conjugated to Alexa Fluor® 488 (1:200, Thermo Fisher Scientific, S11223) to detect biotinylated IB4. In all other experiments, we combined fluorescein-labeled G. simplicifolia lectin, which contains IB4 as the endothelial-specific lectin (1:200, GSL I–BSL I; FL-1101-5, Vector Labs), together with the following secondary antibodies, diluted 1:200 in PBS for 2 hours at RT: Alexa Fluor® 647 AffiniPure® Fab Fragment Donkey Anti-Rabbit IgG (H+L), Cy^TM^3 AffiniPure® F(ab')_2_ Fragment Donkey Anti-Rat IgG (H+L) (Stratech Jackson, 712-166-150), Cy^TM^3 AffiniPure® F(ab')_2_ Fragment

Donkey Anti-Rabbit IgG (H+L) (Stratech Jackson, 711-166-152), and anti-Ki67e660 (50-5698-82, eBioscience/Thermo). After four 15-minute washes in PBS, retinas were mounted on glass slides using Fluoromount-G^TM^ Mounting Medium (00-4958-02, Thermo Fisher Scientific) for P7 and P21 retinas and VECTASHIELD^®^ Vibrance^TM^ Antifade Mounting Medium (H-1700-10, 2BScientific) for all other experiments.

### Tamoxifen treatments

Tamoxifen powder (Sigma, T5648) was dissolved in vegetable oil (peanut oil, Sigma P2144, or corn oil, Sigma C8267) by agitation at 37 °C for 2 h. To activate CreER during the perinatal period, mice received two intraperitoneal injections of 25 µL vegetable oil or tamoxifen (2, 4 or 6 mg/mL) on postnatal day (P) 2 and P4, and eyes were collected on P5, P7, P15, P17 or P21. For experiments assessing the effects of a single tamoxifen injection, mice received one 25 µL tamoxifen (4 mg/mL) intraperitoneal injection on P4, and eyes were collected on P5. To activate CreER in adult mice (6–8 weeks old), 100 µL tamoxifen (10 mg/mL) was administered via intraperitoneal injections once daily for three consecutive days, and eyes were collected 24 h after the final injection. Littermate animals lacking CreER received intraperitoneal tamoxifen or vehicle (corn oil) injections to serve as controls. Both male and female mice were included in all analyses.

### Image acquisition and analysis

IB4-stained P7 retinas were imaged using an SZX16 fluorescent stereomicroscope (Olympus) equipped with a C4742-95 camera (Hamamatsu). Retinal radius and retinal vascular extension were measured in each leaflet using the FIJI (NIH Bethesda) and averaged by retina. Retinal radius was defined as the distance from the optic disc to the retinal margin, whereas retinal vascular extension was defined as the distance from the optic disc to the front of the vascular network divided by the retinal radius. Vascular branchpoints were quantified in 4 fields of view per retina (150 × 150 pixel each) behind the angiogenic front in a region between an artery and a vein using Angiotool v.0.9226 (NIH Bethesda) and averaged by retina. IB4-stained P7, P15, P17 and P21 as well as P5 retinas co-stained for ERG, pHH3, Ki-67 and P21 imaged using a widefield inverted Ti2 fluorescent microscope (Nikon) equipped with a DS-Qi2 camera (Nikon). The entire leaflet (or whole retina for ERG or IB4) was analyzed using FIJI. Vascular area percentage was measured in P7, P15, P17 and P21 retinas by thresholding IB4 signal. For P5 retinas analysis, background fluorescence was subtracted using a rolling ball algorithm with a radius of 10 pixels for ERG and p21, 30 pixels for Ki-67, and 50 pixels for pHH3 and IB4. The number of positively stained cells was automatically quantified using the “Analyze Particle” function in FIJI after fluorescent signal thresholding and then confirmed by visual inspection of each image. Co-localization of pHH3 + or Ki-67 + cells with IB4 was assessed manually. The values for each leaflet were summed to obtain the value for that retina. Images of cleaved caspase 3-stained P5 retinas were acquired using a Stellaris 5 confocal microscope equipped with LAS X software (Leica) and comprised a 291 µm^2^ region between an artery and a vein. For P5 retinas, z-stacks were acquired beginning at the superficial vascular plexus and extending through the full depth of the vascular network. Z-projections were generated using FIJI. Apoptosis was measured by counting the number of cleaved caspase 3 + cells using the multipoint tool in FIJI, and co-localization with IB4 + vasculature was assessed manually. Endothelial apoptotic events were normalized to vascular area to account for differences in vessel density. The vascular area (percentage IB4 + area) was measured by thresholding IB4 signal in FIJI. Images of IB4-stained P21 retinas were acquired with a Stellaris 5, confocal microscope equipped with LAS X software (Leica) and comprised a 581 µm^2^ region between an artery and a vein, with the scan beginning at the surface of the retina and extending through the ganglion cell layer into the outer plexiform layer. Z-projections of the vasculature were generated using a temporal color-coding method in FIJI. Confocal slices containing the superficial, intermediate or deep vascular plexus were extracted from each z projection and processed separately for quantification of vascular coverage; the number of branchpoints relative to the retinal area was determined using Angiotool v0.9226 (NIH Bethesda) in four regions of interest (ROI) for P21 and one ROI for adults. When four ROIs were acquired per retina, it was one for each leaflet, and the values were averaged for each plexus in each retina.

### Gene expression analysis

Total RNA was isolated from P5 retinas using the RNeasy Mini Kit (Qiagen Inc.) and reverse-transcribed using the Superscript IV reverse transcription kit (Thermo Fisher Scientific). cDNA was analyzed by real-time PCR on the QuantStudio 6 Flex Real-Time PCR system (Applied Biosystems) using the SYBR Green master mix (Applied Biosystems) and oligonucleotide primer pairs specific for *Erg* (forward 5’-CCGGATACTGTGGGGATGAG-3’ and reverse 5’-TCTGCGCTCATTTGTGGTCA-3’), *Cdkn1a* (forward 5’-TCGCTGTCTTGCACTCTGGTGT-3’ and reverse 5’-CCAATCTGCGCTTGGAGTGATAG-3’), *mKi67* (forward 5’-GAGGAGAAACGCCAACCAAGAG-3’ and reverse 5’-TTTGTCCTCGGTGGCGTTATCC-3’) and *Actb* (forward, 5’-CACCACACCTTCTACAATGAG-3’ and reverse 5’-GTCTCAAACATGATCTGGGTC-3’). Expression of each target gene was normalized to *Actb* levels, and quantification was performed using the 2^−ΔΔCT^ method [48]. Values are expressed as fold change in the CreER+ relative to the CreER− control group.

### Statistical analysis and reproducibility

One retina from one mouse was considered one biological replicate, whereby both retinas were used for different analyses (e.g., one for staining and the other for gene expression). We analyzed 4 different fields of view from each retina and averaged the values to obtain the value for that sample. Blinding was used during treatment and outcome assessment, with genotypes being disclosed only during statistical analysis. Samples or data points were excluded only in the case of technical equipment or human error that caused a sample to be poorly controlled. Statistical analyses were performed using Prism 10 (GraphPad Software Inc.) or Stata (version 18, StataCorp, College Station, Texas, USA). Data are shown as means ± SD. A non-parametric Mann–Whitney test was performed for all analyses. Interaction between tamoxifen doses and sex was tested by comparing the goodness-of-fit of two linear regression models: a reduced model including only the main effects of treatment and sex, and a full model that also included the treatment-by-sex interaction term. We then used a likelihood-ratio test to test if the full model offered a better fit to the data than the reduced model. Significance was established at *P* < 0.05. *P* values are indicated in each Figure as *P < 0.05, **P < 0.01, ***P < 0.001, ****P < 0.0001. At least two independent litters were used for each analysis to ensure reproducibility and robustness of findings. Sample size estimates for vascular extension as the primary outcome were based on the number of animals needed to observe an effect size of 2.0 standard deviation units (a mean difference of 0.1 and a common SD of 0.05), based on prior published data [13]; using calculations with a 2-sided alpha 0.05 and 80% power, the required sample size was calculated to be seven mice per group.

## Supplementary Information

Below is the link to the electronic supplementary material.

Supplementary file1 (PDF 6241 kb)

## Data Availability

No datasets were generated or analysed during the current study.

## Abbreviation

CreER: Cre recombinase estrogen fusion protein with ligand binding mutation

## References

[1] Payne S, De Val S, Neal A (2018) Endothelial-specific Cre mouse models. Arterioscler Thromb Vasc Biol 38(11):2550–2561. 10.1161/ATVBAHA.118.309669 30354251 10.1161/ATVBAHA.118.309669 PMC6218004

[2] Feil R, Brocard J, Mascrez B, LeMeur M, Metzger D, Chambon P (1996) Ligand-activated site-specific recombination in mice. Proc Natl Acad Sci USA 93(20):10887–10890. 10.1073/pnas.93.20.10887 8855277 10.1073/pnas.93.20.10887 PMC38252

[3] Pitulescu ME, Schmidt I, Benedito R, Adams RH (2010) Inducible gene targeting in the neonatal vasculature and analysis of retinal angiogenesis in mice. Nat Protoc 5(9):1518–1534. 10.1038/nprot.2010.113 20725067 10.1038/nprot.2010.113

[4] Rashbrook VS, Brash JT, Ruhrberg C (2022) Cre toxicity in mouse models of cardiovascular physiology and disease. Nat Cardiovasc Res 1(9):806–816. 10.1038/s44161-022-00125-6 37692772 10.1038/s44161-022-00125-6 PMC7615056

[5] Aspalter IM, Gordon E, Dubrac A, Ragab A, Narloch J, Vizan P et al (2015) Alk1 and Alk5 inhibition by Nrp1 controls vascular sprouting downstream of Notch. Nat Commun 6:7264. 10.1038/ncomms8264 26081042 10.1038/ncomms8264 PMC4557308

[6] Tammela T, Zarkada G, Nurmi H, Jakobsson L, Heinolainen K, Tvorogov D et al (2011) VEGFR-3 controls tip to stalk conversion at vessel fusion sites by reinforcing Notch signalling. Nat Cell Biol 13(10):1202–1213. 10.1038/ncb2331 21909098 10.1038/ncb2331 PMC3261765

[7] Benedito R, Rocha SF, Woeste M, Zamykal M, Radtke F, Casanovas O et al (2012) Notch-dependent VEGFR3 upregulation allows angiogenesis without VEGF-VEGFR2 signalling. Nature 484(7392):110–114. 10.1038/nature10908 22426001 10.1038/nature10908

[8] Chappell JC, Darden J, Payne LB, Fink K, Bautch VL (2019) Blood vessel patterning on retinal astrocytes requires endothelial Flt-1 (VEGFR-1). J Dev Biol 7(3):18. 10.3390/jdb7030018 31500294 10.3390/jdb7030018 PMC6787756

[9] Ho VC, Duan LJ, Cronin C, Liang BT, Fong GH (2012) Elevated vascular endothelial growth factor receptor-2 abundance contributes to increased angiogenesis in vascular endothelial growth factor receptor-1-deficient mice. Circulation 126(6):741–752. 10.1161/CIRCULATIONAHA.112.091603 22753193 10.1161/CIRCULATIONAHA.112.091603 PMC3442373

[10] Serra H, Chivite I, Angulo-Urarte A, Soler A, Sutherland JD, Arruabarrena-Aristorena A et al (2015) PTEN mediates Notch-dependent stalk cell arrest in angiogenesis. Nat Commun 6:7935. 10.1038/ncomms8935 26228240 10.1038/ncomms8935 PMC5426521

[11] Okabe K, Kobayashi S, Yamada T, Kurihara T, Tai-Nagara I, Miyamoto T et al (2014) Neurons limit angiogenesis by titrating VEGF in retina. Cell 159(3):584–596. 10.1016/j.cell.2014.09.025 25417109 10.1016/j.cell.2014.09.025

[12] Claxton S, Kostourou V, Jadeja S, Chambon P, Hodivala-Dilke K, Fruttiger M (2008) Efficient, inducible Cre-recombinase activation in vascular endothelium. Genesis 46(2):74–80. 10.1002/dvg.20367 18257043 10.1002/dvg.20367

[13] Brash JT, Bolton RL, Rashbrook VS, Denti L, Kubota Y, Ruhrberg C (2020) Tamoxifen-activated CreERT impairs retinal angiogenesis independently of gene deletion. Circ Res 127(6):849–850. 10.1161/CIRCRESAHA.120.317025 32635822 10.1161/CIRCRESAHA.120.317025 PMC7447161

[14] Koo Y, Barry DM, Xu K, Tanigaki K, Davis GE, Mineo C et al (2016) Rasip1 is essential to blood vessel stability and angiogenic blood vessel growth. Angiogenesis 19(2):173–190. 10.1007/s10456-016-9498-5 26897025 10.1007/s10456-016-9498-5 PMC4808411

[15] Zhang S, Liu W, Yang Y, Sun K, Li S, Xu H et al (2019) TMEM30A deficiency in endothelial cells impairs cell proliferation and angiogenesis. J Cell Sci 132(7):5052. 10.1242/jcs.225052 10.1242/jcs.225052 30814335

[16] Kim J, Oh WJ, Gaiano N, Yoshida Y, Gu C (2011) Semaphorin 3E-Plexin-D1 signaling regulates VEGF function in developmental angiogenesis via a feedback mechanism. Genes Dev 25(13):1399–1411. 10.1101/gad.2042011 21724832 10.1101/gad.2042011 PMC3134083

[17] Thyagarajan B, Guimaraes MJ, Groth AC, Calos MP (2000) Mammalian genomes contain active recombinase recognition sites. Gene 244(1–2):47–54. 10.1016/s0378-1119(00)00008-1 10689186 10.1016/s0378-1119(00)00008-1

[18] Pugach EK, Richmond PA, Azofeifa JG, Dowell RD, Leinwand LA (2015) Prolonged Cre expression driven by the alpha-myosin heavy chain promoter can be cardiotoxic. J Mol Cell Cardiol 86:54–61. 10.1016/j.yjmcc.2015.06.019 26141530 10.1016/j.yjmcc.2015.06.019 PMC4558343

[19] Semprini S, Troup TJ, Kotelevtseva N, King K, Davis JR, Mullins LJ et al (2007) Cryptic loxP sites in mammalian genomes: genome-wide distribution and relevance for the efficiency of BAC/PAC recombineering techniques. Nucleic Acids Res 35(5):1402–1410. 10.1093/nar/gkl1108 17284462 10.1093/nar/gkl1108 PMC1865043

[20] Wang X, Lauth A, Wan TC, Lough JW, Auchampach JA (2020) Myh6-driven Cre recombinase activates the DNA damage response and the cell cycle in the myocardium in the absence of loxP sites. Dis Model Mech 13(12):6375. 10.1242/dmm.046375 10.1242/dmm.046375 PMC7758623 33106234

[21] Loonstra A, Vooijs M, Beverloo HB, Allak BA, van Drunen E, Kanaar R et al (2001) Growth inhibition and DNA damage induced by Cre recombinase in mammalian cells. Proc Natl Acad Sci USA 98(16):9209–9214. 10.1073/pnas.161269798 11481484 10.1073/pnas.161269798 PMC55399

[22] Ruhrberg C, Bautch VL (2013) Neurovascular development and links to disease. Cell Mol Life Sci 70(10):1675–1684. 10.1007/s00018-013-1277-5 23475065 10.1007/s00018-013-1277-5 PMC3632722

[23] Stahl A, Connor KM, Sapieha P, Chen J, Dennison RJ, Krah NM et al (2010) The mouse retina as an angiogenesis model. Invest Ophthalmol Vis Sci 51(6):2813–2826. 10.1167/iovs.10-5176 20484600 10.1167/iovs.10-5176 PMC2891451

[24] Fruttiger M (2007) Development of the retinal vasculature. Angiogenesis 10(2):77–88. 10.1007/s10456-007-9065-1 17322966 10.1007/s10456-007-9065-1

[25] Fantin A, Ruhrberg C (2015) The embryonic mouse hindbrain and postnatal retina as in vivo models to study angiogenesis. Methods Mol Biol 1332:177–188. 10.1007/978-1-4939-2917-7_13 26285754 10.1007/978-1-4939-2917-7_13

[26] Ticli G, Cazzalini O, Stivala LA, Prosperi E (2022) Revisiting the function of p21(CDKN1A) in DNA repair: the influence of protein interactions and stability. Int J Mol Sci 23(13):7058. 10.3390/ijms23137058 35806061 10.3390/ijms23137058 PMC9267019

[27] Alsina-Sanchis E, Mulfarth R, Moll I, Mogler C, Rodriguez-Vita J, Fischer A (2021) Intraperitoneal oil application causes local inflammation with depletion of resident peritoneal macrophages. Mol Cancer Res 19(2):288–300. 10.1158/1541-7786.MCR-20-0650 33139505 10.1158/1541-7786.MCR-20-0650

[28] Montenegro MF, Pessa LR, Gomes VA, Desta Z, Flockhart DA, Tanus-Santos JE (2009) Assessment of vascular effects of tamoxifen and its metabolites on the rat perfused hindquarter vascular bed. Basic Clin Pharmacol Toxicol 104(5):400–407. 10.1111/j.1742-7843.2009.00377.x 19413660 10.1111/j.1742-7843.2009.00377.x

[29] Ye R, Wang QA, Tao C, Vishvanath L, Shao M, McDonald JG et al (2015) Impact of tamoxifen on adipocyte lineage tracing: inducer of adipogenesis and prolonged nuclear translocation of Cre recombinase. Mol Metab 4(11):771–778. 10.1016/j.molmet.2015.08.004 26629402 10.1016/j.molmet.2015.08.004 PMC4632120

[30] Fantin A, Lampropoulou A, Gestri G, Raimondi C, Senatore V, Zachary I et al (2015) NRP1 regulates CDC42 activation to promote filopodia formation in endothelial tip cells. Cell Rep 11(10):1577–1590. 10.1016/j.celrep.2015.05.018 26051942 10.1016/j.celrep.2015.05.018 PMC4528263

[31] Hayashi S, McMahon AP (2002) Efficient recombination in diverse tissues by a tamoxifen-inducible form of Cre: a tool for temporally regulated gene activation/inactivation in the mouse. Dev Biol 244(2):305–318. 10.1006/dbio.2002.0597 11944939 10.1006/dbio.2002.0597

[32] Chen D, Rukhlenko OS, Coon BG, Joshi D, Chakraborty R, Martin KA et al (2024) VEGF counteracts shear stress-determined arterial fate specification during capillary remodeling. bioRxiv. 10.1101/2024.01.23.57692039803508

[33] Schlecht A, Leimbeck SV, Jagle H, Feuchtinger A, Tamm ER, Braunger BM (2017) Deletion of endothelial transforming growth factor–β signaling leads to choroidal neovascularization. Am J Pathol 187(11):2570–2589. 10.1016/j.ajpath.2017.06.018 28823871 10.1016/j.ajpath.2017.06.018

[34] Gabel F, Aubry AS, Hovhannisyan V, Chavant V, Weinsanto I, Maduna T et al (2020) Unveiling the impact of morphine on tamoxifen metabolism in mice in vivo. Front Oncol 10:25. 10.3389/fonc.2020.00025 32154159 10.3389/fonc.2020.00025 PMC7046683

[35] Zhong Q, Zhang C, Zhang Q, Miele L, Zheng S, Wang G (2015) Boronic prodrug of 4-hydroxytamoxifen is more efficacious than tamoxifen with enhanced bioavailability independent of CYP2D6 status. BMC Cancer 15:625. 10.1186/s12885-015-1621-2 26354796 10.1186/s12885-015-1621-2 PMC4563833

[36] Birdsey GM, Dryden NH, Amsellem V, Gebhardt F, Sahnan K, Haskard DO et al (2008) Transcription factor Erg regulates angiogenesis and endothelial apoptosis through VE-cadherin. Blood 111(7):3498–3506. 10.1182/blood-2007-08-105346 18195090 10.1182/blood-2007-08-105346 PMC2275018

[37] Watson EC, Koenig MN, Grant ZL, Whitehead L, Trounson E, Dewson G et al (2016) Apoptosis regulates endothelial cell number and capillary vessel diameter but not vessel regression during retinal angiogenesis. Development 143(16):2973–2982. 10.1242/dev.137513 27471260 10.1242/dev.137513

[38] Hans F, Dimitrov S (2001) Histone H3 phosphorylation and cell division. Oncogene 20(24):3021–3027. 10.1038/sj.onc.1204326 11420717 10.1038/sj.onc.1204326

[39] Gerdes J, Lemke H, Baisch H, Wacker H-H, Schwab U, Stein H (1984) Cell cycle analysis of a cell proliferation-associated human nuclear antigen defined by the monoclonal antibody Ki-67. J Immunol 133(4):1710–1715 6206131

[40] Garcia-Gonzalez I, Rocha SF, Hamidi A, Garcia-Ortega L, Regano A, Sanchez-Muñoz MS et al (2024) iSuRe-HadCre is an essential tool for effective conditional genetics. Nucleic Acids Res 52(13):e56–e56. 10.1093/nar/gkae472 38850155 10.1093/nar/gkae472 PMC11260470

[41] Biswas S, Shahriar S, Bachay G, Arvanitis P, Jamoul D, Brunken WJ et al (2024) Glutamatergic neuronal activity regulates angiogenesis and blood-retinal barrier maturation via Norrin/β-catenin signaling. Neuron 112(12):1978-1996e1976. 10.1016/j.neuron.2024.03.011 38599212 10.1016/j.neuron.2024.03.011 PMC11189759

[42] Zarkada G, Heinolainen K, Makinen T, Kubota Y, Alitalo K (2015) VEGFR3 does not sustain retinal angiogenesis without VEGFR2. Proc Natl Acad Sci USA 112(3):761–766. 10.1073/pnas.1423278112 25561555 10.1073/pnas.1423278112 PMC4311859

[43] Janbandhu VC, Moik D, Fassler R (2014) Cre recombinase induces DNA damage and tetraploidy in the absence of loxP sites. Cell Cycle 13(3):462–470. 10.4161/cc.27271 24280829 10.4161/cc.27271 PMC3956542

[44] Sahasrabuddhe V, Ghosh HS (2022) Cx3Cr1-Cre induction leads to microglial activation and IFN-1 signaling caused by DNA damage in early postnatal brain. Cell Rep 38(3):110252. 10.1016/j.celrep.2021.110252 35045285 10.1016/j.celrep.2021.110252

[45] Higashi AY, Ikawa T, Muramatsu M, Economides AN, Niwa A, Okuda T et al (2009) Direct hematological toxicity and illegitimate chromosomal recombination caused by the systemic activation of CreERT2. J Immunol 182(9):5633–5640. 10.4049/jimmunol.0802413 19380810 10.4049/jimmunol.0802413

[46] Pontes-Quero S, Fernandez-Chacon M, Luo W, Lunella FF, Casquero-Garcia V, Garcia-Gonzalez I et al (2019) High mitogenic stimulation arrests angiogenesis. Nat Commun 10(1):2016. 10.1038/s41467-019-09875-7 31043605 10.1038/s41467-019-09875-7 PMC6494832

[47] Madisen L, Zwingman TA, Sunkin SM, Oh SW, Zariwala HA, Gu H et al (2010) A robust and high-throughput Cre reporting and characterization system for the whole mouse brain. Nat Neurosci 13(1):133–140. 10.1038/nn.2467 20023653 10.1038/nn.2467 PMC2840225

[48] Livak KJ, Schmittgen TD (2001) Analysis of relative gene expression data using real-time quantitative PCR and the 2(-delta delta C(T)) method. Methods 25(4):402–408. 10.1006/meth.2001.1262 11846609 10.1006/meth.2001.1262

