# Supplementary material for: CreER activation transiently disrupts angiogenesis by reducing proliferation and promoting apoptosis in vascular endothelial cells
Source: Angiogenesis. 2026 May 5;29(3):32. doi: 10.1007/s10456-026-10040-0 (PMC13144190; doi:10.1007/s10456-026-10040-0)

Supplemental data

Supplemental Fig. S1

CreER toxicity affects body weight gain and retinal vascular extension in neonatal mice independently of tamoxifen and loxP sites.

(**A**) Schematic representation of the regime used to treat wild-type C57BL6/J pups with 25 µL vehicle or the indicated doses of tamoxifen on P2 and P4, and quantification of body weight and retinal extension relative to retinal radius on P7 (n = 8-14 pups per group). (**B**) Schematic representation of the treatment of *Cagg*-CreER+ and *Cagg*-CreER- pups carrying the *Rosa26*^{tdTom} reporter with 100 µg tamoxifen on P2 and P4, and retina staining on P5 with IB4 (shown in inverted grey scale) and antibodies for RFP (shown in green); the endogenous tdTom fluorescence signal is shown in red. Scale bars: 50 µm. (**C**) Schematic representation of the treatment of *Cagg*-CreER+ and CreER- pups carrying or not carrying the *Rosa26*^{tdTom} reporter with the indicated tamoxifen doses on P2 and P4; retinas were stained for IB4 on P7 to assess vascular extension; n = 5-14 pups per group; samples from tdTom+ (dark red) and tdTom- (blue) mice. Values from CreER+ samples were normalized to the average of the values for the CreER- group. Data are shown as mean ± SD. Each data point represents one retina from one mouse. ***P< 0.001; ****P<0.0001. Mann-Whitney U test.

Supplemental Fig. S2

Retinal vasculature progressively normalizes after CreER activation.

Cagg-CreER+ and *Cagg*-CreER- littermate pups on a C57BL6/J background were treated with 100 µg tamoxifen on P2 and P4, and analyzed on P15, P17 or P21 after staining for IB4. (**A**) Schematic representation of the tamoxifen treatment. (**B**) Representative images of P15, P17 and P21 CreER+ and CreER- flat-mounted whole retinas and a higher magnification of a representative leaflet, after staining with IB4 (shown in greyscale). (**C**) Quantification of vascular coverage (n = 3-7 pups per group). For comparison, the quantification of vascular coverage at P7 based on images generated for vascular extension in Fig. 1, was included in the quantification. Data are shown as mean ± SD. Each data point represents one retina from one mouse. **P< 0.01; ****P<0.0001; ns: not significant (P>0.05). Mann-Whitney U test. Scale bars: 500 µm.

Supplemental Fig. S3

Increased number of retinal non-endothelial cells in mitosis after *Cagg*-CreER activation.

(**A-D**) Schematic representation of the regime used to treat *Cagg*-CreER+ and *Cagg*-CreER- littermate pups on a C57BL6/J background with 100 µg tamoxifen on P4, referred to as the tam (1) condition, or on P2 and P4, referred to as the tam (2) condition, and then analyzed on P5. (**A,B**) Body weight for the (**A**) tam (1) and (**B**) tam (2) condition (n= 16 per group). (**C**) Quantification of cleaved caspase+ cells detected outside of the IB4+ vasculature per retina using images generated for endothelial cell analysis in Fig. 3 (n = 5-6 per group). (**D,E**) Representative images of P5 CreER- flat-mounted retinas from pups receiving 100 µg tamoxifen on P2 and P4 labeled with IB4 (shown in inverted grey scale) and the anti-Ki67 antibodies SP6 (abcam, #ab16667) that detects Ki67^{high} cells or SolA15 eFluor™ 660 (eBioscience™, #50-5698-82) that detects Ki67^{high} and Ki67^{low} cells. In D), both antibodies are used together, and in (**E**), the SP6 antibody is used together with an antibody for pHH3. Scale bars: 100 µm. (**F**) Schematic representation of the regime used to treat *Cagg*-CreER+ and *Cagg*-CreER- littermate pups on a C57BL6/J background with 100 µg tamoxifen on P2 and P4, referred to as the tam (2) condition and quantification of the number of Ki67^{high} and pHH3+ cells detected outside of the IB4+ vasculature per retina using images generated for endothelial cell

analysis in Fig. 4 (n = 5-6 per group). Data are shown as mean ± SD. Each data point represents one mouse and one retina from one mouse. $^{**}P < 0.01$; ns: not significant ($P>0.05$). Mann-Whitney U test.

Supplemental Fig. S4

Endothelial cell selectivity of p21 upregulation in the retina after *Cagg*-CreER activation.

Schematic representation of the regime used to treat *Cagg*-CreER+ and *Cagg*-CreER- littermate pups on a C57BL6/J background with 100 µg tamoxifen on P2 and P4, referred to as the tam (2) condition. Retinas were analyzed on P5. Representative images of P5 retinas stained with IB4 (shown in blue) and antibodies for pHH3 (shown in green) and p21 (shown in pink) illustrate endothelial selectivity of p21 upregulation in the retina. Scale bars: 100 µm.

Supplemental Fig. S5

No p21 upregulation in angiogenic endothelium after vehicle treatment, but 24 hours after a single tamoxifen injection.

(**A,B**) *Cagg*-CreER+ and *Cagg*-CreER- littermate pups on a C57BL6/J background were treated with 2 doses of vehicle (**A**) or one dose of 100 µg tamoxifen (**B**) or two doses of tamoxifen (**C,D**), referred to as the tam (1) and tam (2) conditions, respectively, before retinas were labeled with IB4 and stained with antibodies for p21. In (**A,B**), P5 retinas are shown with IB4 in inverted greyscale and p21 in magenta. In (**C,D**), images of all three retinal plexi are shown as maximum projection confocal z-stacks and used for quantification of the number of p21+ cells per retina (n = 3-6 per group). Data are shown as mean ± SD. Each data point represents one retina from one mouse. $^{**}P< 0.01$; ns: not significant ($P>0.05$). Mann-Whitney U test. Scale bars: 100 µm.

Supplemental Figure S1

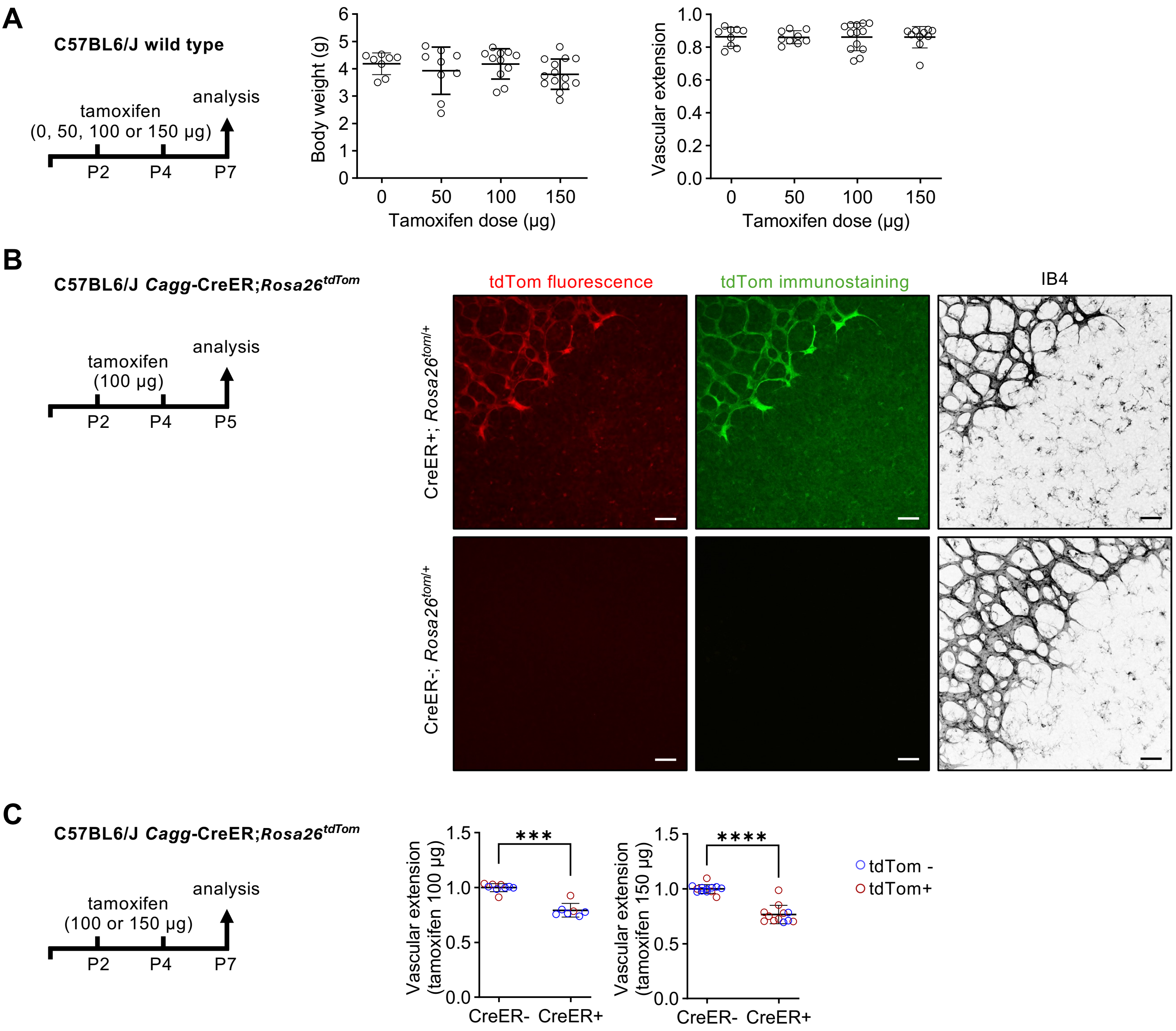

Supplemental Figure S2

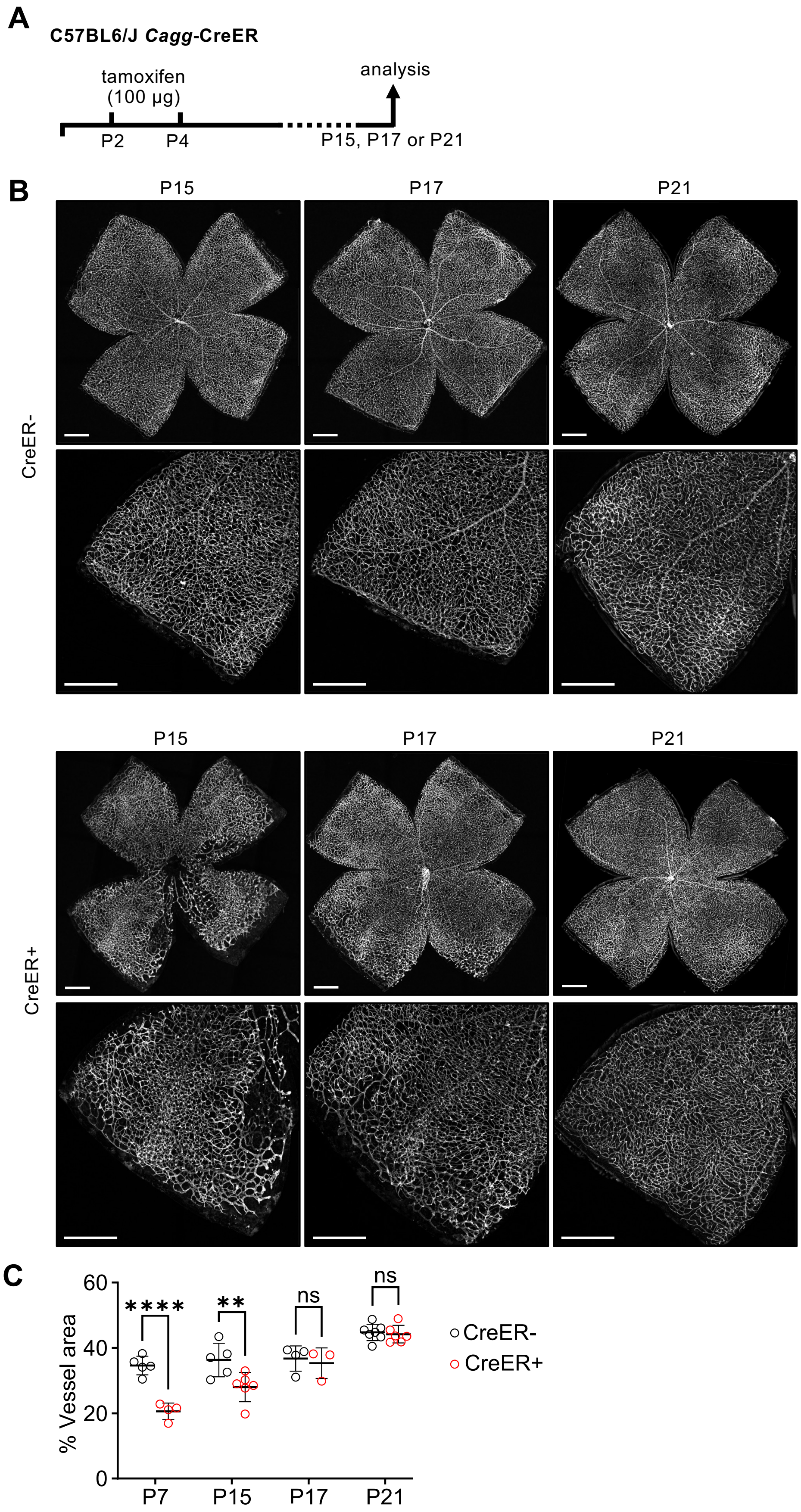

Supplemental Figure S3

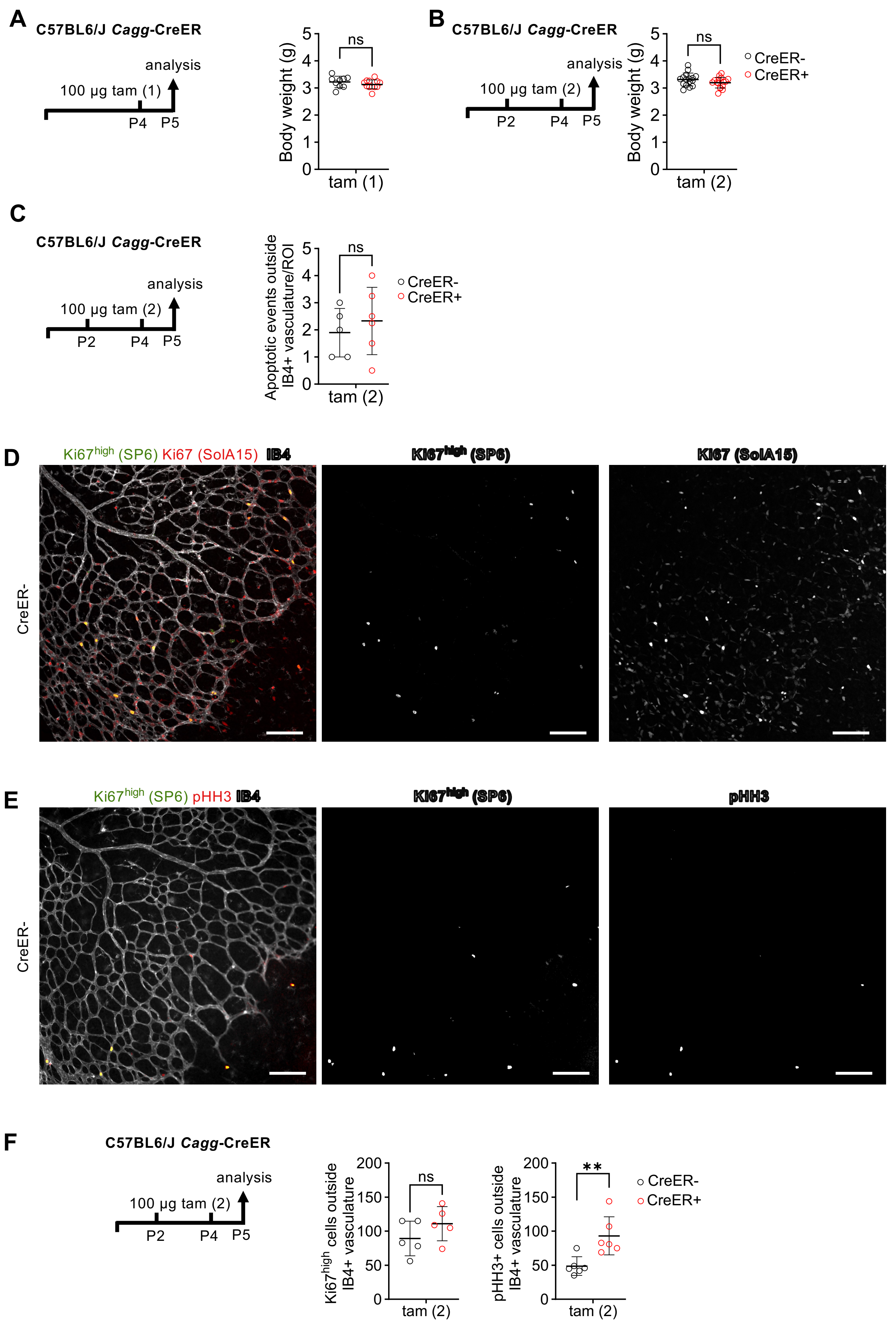

Supplemental Figure S4

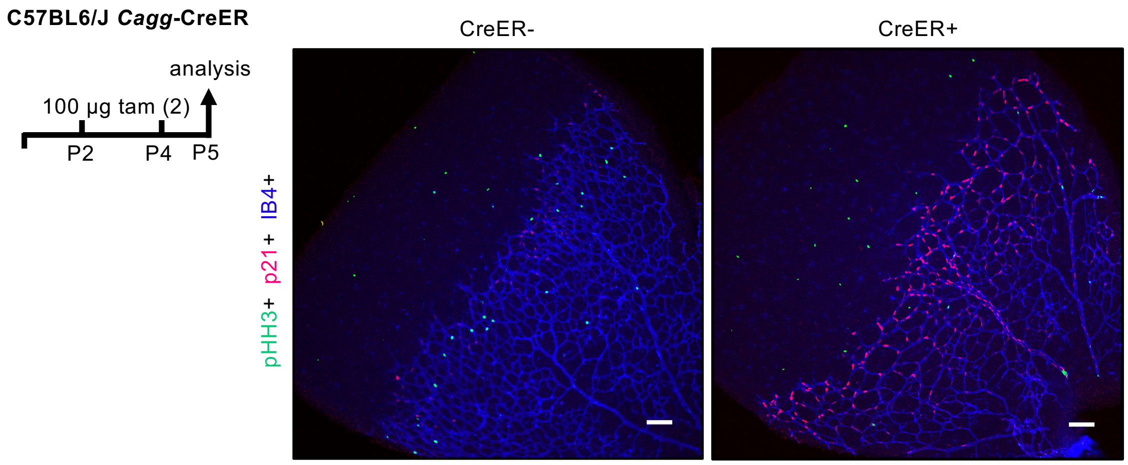

Supplemental Figure S5

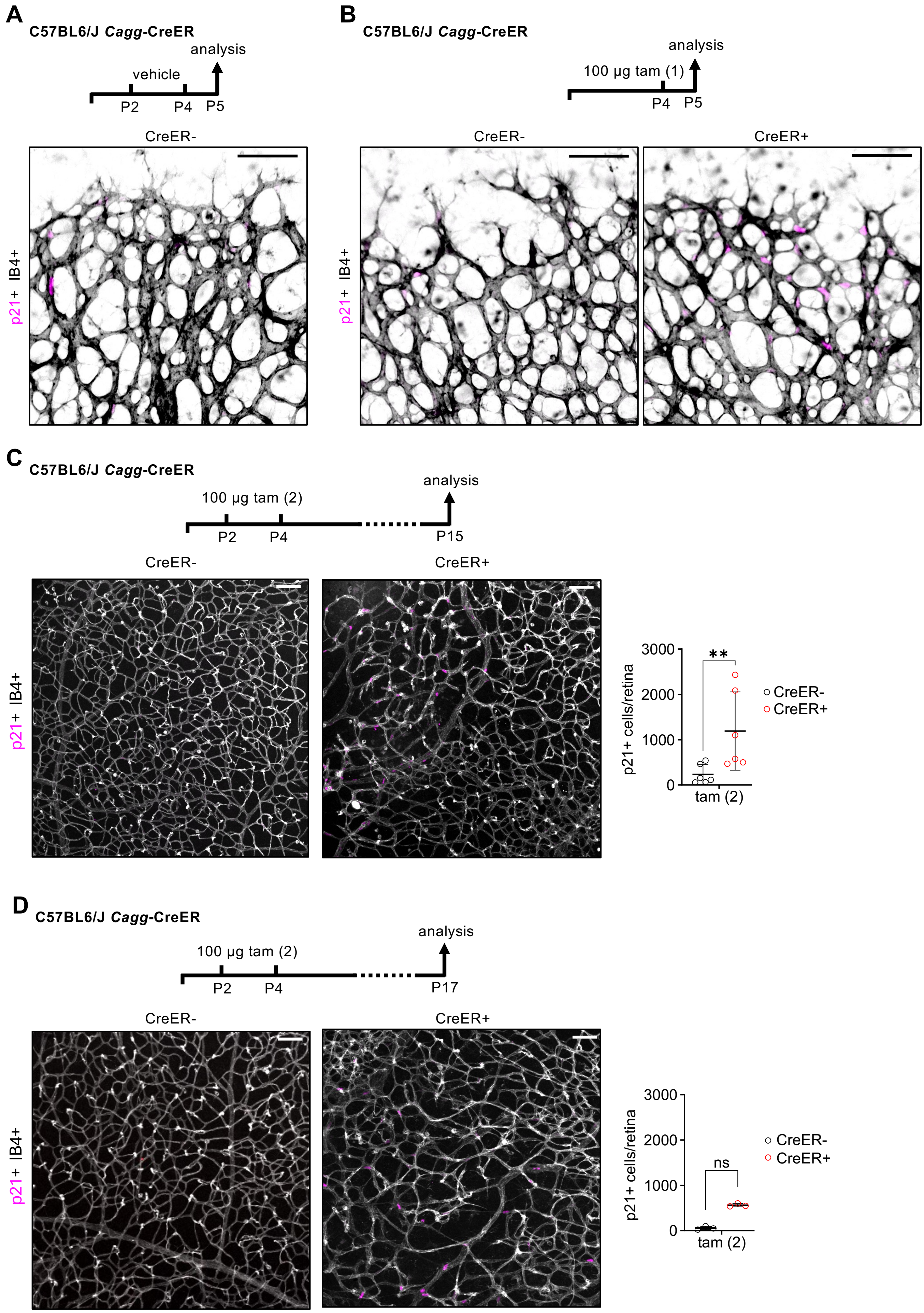

Supplement: Supplementary file 1 — Supplementary file1 (PDF 6241 kb) [file 10456_2026_10040_MOESM1_ESM.pdf]
